# ISFET Based Microsensors for Environmental Monitoring

**DOI:** 10.3390/s100100061

**Published:** 2009-12-24

**Authors:** Cecilia Jimenez-Jorquera, Jahir Orozco, Antoni Baldi

**Affiliations:** Instituto de Microelectrónica de Barcelona, IMB-CNM (CSIC), Campus UAB, 08193 Bellaterra, Barcelona, Spain; E-Mails: jahir.orozco@obs-banyuls.fr (J.O.); antoni.baldi@imb-cnm.csic.es (A.B.)

**Keywords:** ISFETs, microsensors, environmental monitoring

## Abstract

The use of microsensors for in-field monitoring of environmental parameters is gaining interest due to their advantages over conventional sensors. Among them microsensors based on semiconductor technology offer additional advantages such as small size, robustness, low output impedance and rapid response. Besides, the technology used allows integration of circuitry and multiple sensors in the same substrate and accordingly they can be implemented in compact probes for particular applications e.g., *in situ* monitoring and/or on-line measurements. In the field of microsensors for environmental applications, Ion Selective Field Effect Transistors (ISFETs) have a special interest. They are particularly helpful for measuring pH and other ions in small volumes and they can be integrated in compact flow cells for continuous measurements. In this paper the technologies used to fabricate ISFETs and a review of the role of ISFETs in the environmental field are presented.

## 1. Introduction

The field of microfabricated chemical sensors research based on Ion-sensitive Field Effect Transistors (ISFETs) and related sensors has been dynamic since Bergveld introduced the ISFET concept in 1970 [1]. From then many scientific papers as well as some interesting reviews concerning such semiconductor based sensors have been published [2–4]. The initial interest came from the advantages of ISFET over conventional ion-selective electrode (ISE) such as small size and solid-state nature, mass fabrication, short response time and low output impedance. Other features such as the integration of compensation and data processing circuits in the same chip offered also new perspectives for these sensors [5].

Variation of ISFET selectivity, intrinsically sensitive to pH due to the inorganic nature of the gate, was obtained by modifying the gate material or by depositing a selective membrane or a biorecognition element onto the gate. The resulting sensors were called chemically sensitive field effect transistors (CHEMFET). Initially ionic selective ISFETs made use of heterogeneous membranes of silver halides [6] and membranes based on polyvinyl chloride (PVC), exploiting the same technology developed for ISEs [7,8]. Later on, new materials for membrane development were explored to improve the poor adherence of PVC membranes on to the ISFET surface and the low reproducibility due to the manual patterning and deposition membrane methods. Photo-cured polymers, which are compatible with photolithographic techniques, were proposed as a feasible solution due to their enhanced adherence to the silanised gate of ISFET devices. Several polymers were studied for developing ion selective membranes including polysiloxanes [9], polyurethanes [10,11] and other methacrylate derived polymers [12]. ISFETs developed with these new polymers demonstrated higher performances regarding reproducibility and long-term stability compared with those with PVC membranes [13]. The majority of these ionic based ISFETs were applied to environmental and clinical analysis.

CHEMFETs have also been applied for the detection of molecular species using field effect transistors modified with enzymes (ENFET). The response of ENFETs to a certain enzymatic substrate is based on the variation of ions concentration (pH, NH^4+^, CO_3_^2−^) produced or consumed during the catalytic enzyme reaction. Substrates like urea [14–16], glucose [17], penicillin [18] and acetylcholine [19] are the most common analysed. Most of them make use of enzymatic membranes immobilised by adsorption, crosslinking with glutaraldehyde and entrapment in a polymeric membrane. Use of polymeric photocurable membranes for enzyme entrapment is advantageous since it can be patterned at wafer level in the same way as ionic sensitive membranes [6,20]. Those biosensors have been used mainly in environmental applications for pesticide detection.

The application of ISFET based sensors in different areas of analytical chemistry has advanced new technological developments and the implementation of sensors in more automated systems. The main example is their application in continuous flow systems such as Flow Injection Analysis (FIA) and Sequential Injection Analysis (SIA) with key features including the miniaturization of the flow cell, and therefore low reagent consumption and fast analysis throughput, and the minimization of ISFET drift due to the relative signal related to the base-line achieved [21–23]. In a more advanced approach the integration of ISFET based sensors in microsystems for chemical analysis (*i.e.*, the so called Micrototal Analysis Systems, μTAS or lab-on-chip, LoC) is another key feature of these devices mostly for bed-side monitoring and environmental control [24,25].

Currently, the advances in microelectronic technologies have also been exploited for improvement in ISFET fabrication. Apart from the technological considerations of NMOS (n-metal oxide Semiconductor) or CMOS (Complementary Metal Oxide Semiconductor) -based technology used (see Section 2), the correct packaging of ISFET based sensors is critical. All electrical parts of ISFET must be protected leaving only the gate area open to liquid contact. This process has been performed usually with thermosetting resins, thus being totally manual and lowering manufacturing reproducibility and increasing personal costs. For this reason many new alternatives have been developed in order to look for a more automated method for encapsulation. An approach described by Bratov *et al.* [26] proposes the use of photocurable polymers. These polymer layers can be patterned by exposure to UV with a standard mask aligner system thus permitting the semi-automation of the encapsulation process. Besides, the functionalization of the chip surface by means of adhesion promoters with silanol and acrylate or methacrylate functional groups provides better adhesion of these polymeric layers.

Although ISFET history began in the seventies, commercialization of probes with ISFETs only started in the nineties. This fact can be explained due to a few practical limitations related to the inherent properties of the devices, such as drift, temperature and light sensitivity, and technological limitations such as encapsulation and the need for a stable miniaturized reference electrode [13]. Approaches for the development of a reference FET (REFET) by means of the deposition of different kind of layers on top of the ISFET gate to obtain a non-sensitive sensor working as reference electrode have been extensively described in the literature [27–29]. However, all these approaches suffer short lifetime and residual ionic sensitivity. Therefore the solution currently taken has been the miniaturization of a conventional Ag/AgCl reference electrode and even the integration in the silicon substrate [30–31].

Currently pH-ISFET probes are commercially available from several companies all over the world [*i.e.*, ThermoORION (USA), Sentron (NL), Microsens S.A. (CH), Honeywell (USA), D + T Microelectronica (SP)]. Almost all of these probes are used for general laboratory purposes. Therefore they have sizes and designs similar to the traditional ion-selective electrode probe. Some companies are supplying needle-type probes for specific applications mainly in the food industry, for meat, beverages, and even for direct soil monitoring. Analytical instrumentation companies providing ISFET based probes do not fabricate the devices, but they are usually bought from a few foundries specialized in non-standard microelectronic fabrication processes.

In this review a brief description of ISFETs fabrication and technological key points will be exposed. The application of ISFETs in environmental monitoring will be classified according to the different fields of environmental measurement where the use of ISFETs should be advantageous over conventional ion selective electrodes, such as surface and waste waters, soils and crops, and geological barriers monitoring.

## 2. Technologies for ISFETs Fabrication

A large number of fabrication methods for ISFET sensors have been proposed during the last thirty years. When the materials and processing equipment used for ISFET fabrication are common (or do not interfere with) standard Integrated Circuit (IC) technologies, such as CMOS, the fabrication is said to be compatible with such technologies, which means that the ISFETs and ICs can be fabricated in the same clean room. There are different degrees of technological compatibility ranging from specially designed fabrication sequences with non-standard process to unaltered standard technology (including design rules). The former ones allows for a better selection of materials and geometries, which results in devices with optimal chemical and electrical characteristics. The latter ones can facilitate the fabrication in a commercial foundry, which results in lower cost and enables the integration of conditioning circuits on the same chip. Variations in the encapsulation process to fulfil with requests of particular applications are the most commonly developments that are been implemented. In this section, technology of ISFET fabrication and all the improvements carried out in order to overcome inherent drawbacks of the devices and to take advantage of their features have been reviewed. These improvements have also considered aspects like implementation in multisenor probes, miniaturization of probes and integration in automated and flow systems.

**Figure 1.**
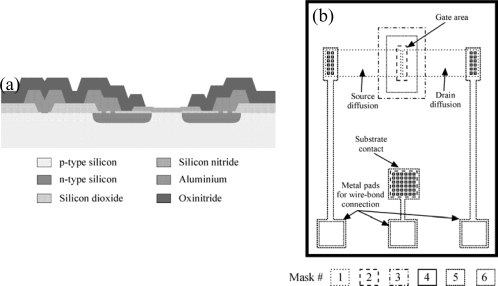
(a) Cross-sectional schematic representation of the NMOS ISFET. (b) Fabrication masks for NMOS ISFETs.

A common technology for ISFET fabrication is based on an Aluminum gate NMOS technology. Following is a description of the fabrication process used at the Instituto de Microelectrónica de Barcelona (IMB-CNM) (see Figure 1). The devices are fabricated on <100> p-type silicon wafers with boron doping of 1.5 × 10^16^ cm^−3^. This doping level ensures low field leakage currents even though no field implant is performed. The process starts with a thick field oxide growth (8,000 Å), which is patterned with mask #1 (source and drain areas). An implant with phosphorous forms the source and drain zones. A second patterning of the field oxide using mask #2 (thin oxide area) and a subsequent thin oxide growth (780 Å) and nitride deposition by Low Pressure Chemical Vapor deposition (LPCVD) (1,000 Å) form the gate double layer dielectric. The nitride is patterned with mask #3 to leave it on the gate area only. Contact openings are then etched (mask #4) followed by deposition and patterning of 0.5 μm aluminum to form metal connections (mask #5). Finally, a 3,000 Å oxide and 4,000 Å nitride, both using Plasma Enhanced CVD are deposited and patterned (mask #6). Figure 1a shows a cross-section of the final structure and Figure 1b a superposition of the fabrication mask set (not to scale).

The drain and source areas are often designed long enough to avoid metal contacts to be close to the gate area, which is exposed to the solution. However, if the passivation layer is a good moisture barrier this condition is not necessary and the overall device dimensions can be reduced significantly by designing devices with short drain and source areas.

Once the ISFET chip is fabricated the process continues with the encapsulation. Encapsulation of ISFETs (and chemical sensors in general) is challenging and often accounts for most of the final cost of the sensor. Technologies or structures that facilitate encapsulation are therefore important to make the sensor production feasible in terms of cost and reliability. Two examples of such structures are back side contacts and trench-isolation. Following is a description of such approaches.

**Figure 2.**
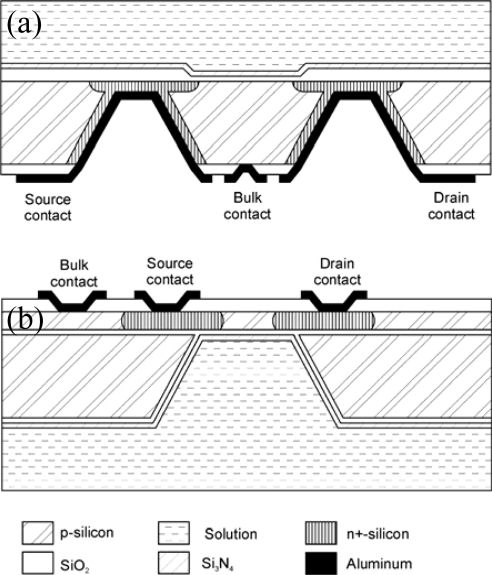
Cross-sectional schematic representation of back-side contacted structures.

Several groups have proposed the development of ISFET chips having the electrical contacts on the opposite side to the chemically sensitive gate (BSC-ISFET, back side contacted ISFET). In this way the electric connections are kept further away from the solution without needing very long chips. Moreover, this approach can yield sensors having a flatter and easier to clean surface since it is not necessary to deposit any encapsulant material in the sensitive side of the chip. Figure 2 show two typical BSC-ISFET structures. Both structures have large cavities in one side so that some areas of the silicon chip are thinner and n-type regions can be formed that contact both sides of the chip. In some cases (Figure 2a) the ISFET gate is shaped on the flat side and the contacts are formed in the cavities [32–35], whereas in other cases (Figure 2b) the contacts are structured in the flat side and the ISFET gate is formed inside a cavity on the other side [36–38]. In order to achieve a better control of the silicon thickness in the cavity area Bond-and-Etch-back Silicon-On-Insulator (BESOI) wafers are often used [33,35–37]. A comprehensive review of BSC ISFETs can be found in [39].

**Figure 3.**
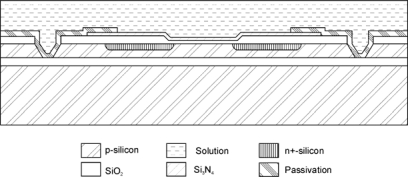
Cross-sectional schematic representation of a trench-isolated NMOS ISFET.

In regular ISFET technologies the chip edges also need to be electrically isolated from the solution in order to avoid instability caused by leakage currents flowing from the substrate to the reference electrode. This complicates the encapsulation procedure, reduces the sensor reliability and limits the minimum width of the chip. Different approaches have been proposed to overcome this problem. One is the fabrication of ISFETs with SOI (Silicon-Oxide-Silicon wafers) technology, in which the transistors are isolated from the substrate [37,40] or the substrate is nonconducting (Silicon on Sapphire wafers) [41,42]. Figure 3 shows a schematic representation of an ISFET fabricated using BESOI wafers with a 5 μm-thick top silicon layer [39]. The fabrication is similar to the metal-gate NMOS with a few additional steps for the formation of isolation trenches.

The fabrication of ISFET with CMOS technologies, allowing sensors and circuits monolithically integrated, has been also used by many research groups [43–56]. In principle, no improvement on signal to noise ratio is expected from this integration, as claimed by Begveld [4,5], since the ISFET works as an impedance transformer at the spot of measurement. However, there are other reasons that make this option interesting. For example, sensors with digital serial output have started to appear in the market and are an attractive option for the implementation of control systems. In this case, the sensor chips require incorporation of conditioning and analog-to-digital conversion (ADC) circuits. Another situation that requires circuit integration arises in applications using large arrays of sensors. An example could be a multi-well plate cell monitoring system for drug screening having ISFET sensors in the bottoms of the single wells [57]. In such application the number of connection pads required to address individual sensors is inconveniently high and some multiplexing circuitry becomes necessary. On chip temperature and drift compensation [43] and storage of calibration data [5] are other examples of applications that could make use of circuit integration next to the sensor.

## 3. ISFETs Applications in the Environmental Field

In order to protect and conserve natural resources, nowadays the directives concerning to water, soil and air quality are very strict. In this context, exigency of international normative has been extended towards the disposure and/or treatment of wastes. For this reason, evaluation and control of quality parameters, not only of environmental matrixes but also of areas dedicated to disposal of wastes are of special interest.

Currently, monitoring is carried out using methodologies and systems based on conventional electrodes and/or standard analytical techniques. Application of ISFET based sensors to environmental monitoring offers valuable advantages compared with ISEs or glass electrodes. In that field, miniaturization in most cases is not critical because provision of sample is almost unlimited. But in some circumstances, *i.e.*, where the sensors have to be packaged in small probes or in microsystems for *in situ* or on-line measurements, ISFETs become competitive due to their small size and robustness.

In this section, applications of ISFETs for monitoring chemical parameters of environmental interest in waters, soils and geochemical barriers are reviewed.

### 3.1. Surface and Waste Waters

Measurement of pH and other parameters (e.g., nitrates, ammonium, pesticides, surfactants) as an indicative of the global quality of water and wastewater is required by Water Agencies Policies. In particular, a range of pH between 6.5 and 8.5 is fixed by regulation agencies for waters addressed to drink. Glass electrodes are used to monitor pH in waters but they lack robustness and suffer from the blocking of the membrane surface when the sample contains a lot of suspension material. In this context, ISFETs are a good alternative to glass electrodes.

#### General water applications

Nitrate is a key parameter for environmental analysis. This ion is present in waters at very low concentrations and the directives according to water quality for drinking uses establish a maximum of 10–25 mg/L, depending on the regulatory agency. Thus, the most critical point of nitrate sensors is the detection limit. Another problem associated with analysis of this ion, mainly for *in situ* analysis, is the presence of interferences, like chloride and nitrite, present also in waters in comparable levels to those of nitrate. Campanella *et al.* [58] reported the use of ISFETs for nitrate analysis in waters using membranes based on a PVC-PVA -poly(vinylalcohol)-PVAc poly(vinyl acetate) copolymer. In this paper, the high selectivity of sensor against common interfering ions such as chloride and nitrite and the wide linear range compared with other CHEMFETs described in the literature was outlined. High recovery data was also obtained with river and soil aqueous extract samples.

Wakida *et al.* [59] developed a prototype of pH and nitrate checker for acid-rain monitoring using Shindengen ISFETs, thus demonstrating good agreement between results and chromatographic methods. pH in single rain droplets was also measured by Poghossian *et al.* [60] and compared with bulk samples measured with a glass electrode.

A few EU funded projects have addressed the application of ISFET based systems to water monitoring. Within the framework of the SEWING project of the 5^th^ Frame Program the development of a cheap and flexible system based on ISFET and CHEMFET for water monitoring was developed. A special flow through chamber containing NH_4_^+^-ISFETs, temperature and reference electrode sensors was described within this project [61].

The most recent FP6 project called WARMER is studying the viability of ISFET based sensors, among others, for environmental monitoring. Here the aim was to fabricate a miniaturized system integrating the sensors, circuitry and software for data conditioning and results visualization. Template-Boyer *et al.* report in [62] the development of pH, NH_4_^+^ and NO_3_^−^ -ISFETs using siloprene membranes for water monitoring.

Although in-field application of sensors should be the ideal way to obtain information in real-time, it generates a lot of problems due to the necessity of frequent sensor calibration and the interferences presented. One attractive alternative is the use of on-line and at-line continuous flow systems. Those systems are able to take the sample automatically and make other processes like calibration, sample treatment and sensor conditioning in a more or less autonomous way. They can be installed near the sampling place (*i.e.*, rivers, lakes, industrial wastes) and can also accomplish some of the requirements for in-field monitoring like low power consumption, high autonomy and robustness. Regarding flow-injection (FI) systems combined with potentiometric sensors [63] additional advantages can be obtained such as high reproducibility due to the controlled dispersion of the sample zone, low sample consumption and fast response. If working with ionic sensors, the operational lifetime of membranes can be increased because of minimal contact time between membrane and sample.

The potentialities of integrating ISFETs into continuous flow systems were outlined early [22,63]. Those systems exploit the small dimensions of devices (low sample dispersion is obtained) and permit the minimization of one of the most important problems of ISFET static measurements, the drift, due to the relative signal to base-line achieved. Moreover, the technological advances in fabrication of monolithic arrays of sensors are attractive for applications in the field of multiparametric analysis requiring small volumes and high sampling rates [64].

One of the most critical aspects of flow systems combined with ISFET sensors is the flow-cell design and the encapsulation of ISFET in a way hydrodynamic characteristics of stream are not changed and a good cleaning of the sensor surface is achieved. Also a minimum volume is preferred.

**Figure 4.**
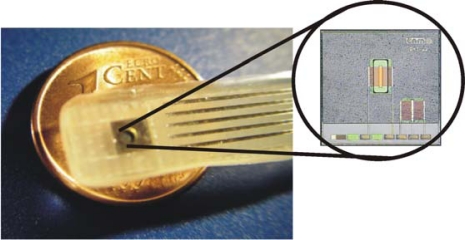
ISFET probe fabricated at IMB-CNM. Photograph of the chip with a pre-encapsulation film.

The introduction of ISFETs in FI systems for environmental applications was early reported by Alegret *et al.* [65]. For that application, an ISFET with a pH-sensitive Si_3_N_4_ gate fabricated at IMB-CNM with a modified aluminium gate NMOS technology was used [13]. Figure 4 shows an ISFET xip pre-encapsulated by means of photocurable polymer [26] and fixed and encapsulated in a Printed Circuit Board (PCB) ready to be used. This encapsulation process and several post-process treatments based on UV-light exposure and chemical cleaning [13] permitted to obtain a reproducible technology. As shown in Table 1 the electrical parameters and chemicals characteristic of 450 encapsulated ISFETs from the same wafer were tested resulting in a yielding of 82%. The devices showing a leakage current over 10 nA or high V_out_ voltage were rejected. Electrical parameters such as Threshold voltage (V_th_) and Transconductance (G_m_) measured at pH 7 were in agreement with estimated values. The average slope from the calibration curve for a range between pH 2 and 12 was 57.0 mV·pH^−1^ (sd 1.4, n = 413) as expected for Si_3_N_4_ ISFETs. The long-term stability of these devices, when measuring in aqueous solutions was estimated between seven months to one year. The drift after pre-conditioning was around 0.5 mV·h^−1^ in a pH 7.0 solution. The temperature coefficient (T.C) measured in a pH 7.0 buffer solution and with Id = 100 μA was around −1.0 mV·°C^−1^.

A flow system for ammonium analysis in waters using the described pH ISFET and a gas diffusion assembly was proposed [65]. The ISFET was implemented in a flow cell fabricated with methacrylate and sandwich configuration. With this system, a linear response in a range from 10^−4^ to 10^−2^ mol·L^−1^ of NH_4_^+^ and a high reproducibility of 0.5% was obtained. A sampling rate of 40–50 h^−1^ was achieved. This flow cell was later on improved by incorporating a nitrate solid-state ion selective electrode (ISE) based on composite technology in the flow assembly. This ISE was acting as a reference electrode with the purpose to compact the detector cell –ISFET and reference electrode- and to avoid junction potential problems associated with usual reference electrodes [21]. This system was used for measuring pH, showing a sampling rate of 140 h^−1^ and a reproducibility of 0.5%

The use of ISFETs with back-side contacts (BSC-ISFETs) was claimed advantageous for dynamic applications compared to front-side contacted ISFETs. Firstly, the encapsulation process is more suitable for mass-production. Secondly, the electrical parts are better protected against chemical environment and finally, the flat surface exposed to the solution stream – there is no encapsulation film hindering the formation of a stagnant surface – help to obtain favorable hydrodynamic performances. Alegret *et al.* reported a FI system with a BSC-ISFET to monitor ammonium ions in waters [66]. It used a NH_4_^+^ ISFET with a PVC membrane and a gas dialysis module to separate the ammonium gas from the sample, thus avoiding common interferences for NH_4_^+^ selective membrane. The gas formed in the donor solution diffused across the permeable membrane and was collected by a suitable acceptor solution.

A more advanced assembly for on-line water monitoring using BSC-ISFETs was described by Jimenez *et al.* [67]. The flow cell, with an internal volume of 50 μL and fabricated with Teflon, contained a screw-type probe for the ISFET as well as the same probe design for a Ag/AgCl reference electrode fabricated using standard technology. This system was applied for monitoring pH, NH_4_^+^, Ca^2+^ and NO_3_^−^ using a modular design. An analysis rate of 20 h^−1^ was estimated for all parameters. Although ionic membranes used were based on PVC, the long-term stability of sensors was quite satisfactory—one month for pH sensor and several weeks for ionic sensors under continuous working.

On-line monitoring of biological parameters of waters is also a basic requirement for environmental control. A system reported by Cambiaso *et al.* [68] monitored the presence of living micro-organisms by measuring cell-induced acidification rates with a pH-ISFET in microsamples of water using a specially designed flow-through micro-chamber. *Escherichia coli* was used as pattern microorganism. Although low precision was achieved, the usefulness of the system as an alarm device for detecting the presence of micro-organisms in waters was outlined.

Recent advances on environmental applications are related to new methods and circuitry associated with ISFETs. An analog processor design for an ISFET based flow system and its application in smart living space was presented by Chung *et al.* [69]. The authors stated that the results could be directly used in drinking water and swimming pool monitoring for improving living space and quality. On the other hand, a multisensor system including ISFETs, a bridge-type constant voltage circuit, and temperature compensation circuitries was developed to detect pH and chloride ions for water quality monitoring applications. This design offers a sensitivity of over 54 mV/pH and an improved temperature coefficient (T.C.) of 0.02 mV/°C [70].

Recently, Chen *et al.* presented a new intelligent ISFET sensor system based on a commercial ISFET from D + T Microelectrónica (Spain) with temperature and drift compensation for long-term monitoring [71]. Measurements reported suggest that the ISFET system using novel compensation can provide significant immunity against voltage and temperature drift, which are favourable towards robust measurements in environmental monitoring applications.

#### Monitoring of wastewater

Portable analytical systems are being increasingly deployed in industrial water collectors or wastewater treatment plants to evaluate and control water quality parameters. As mentioned above, most of them are based on conventional and standard analytical techniques. The use of solids state sensor arrays and microsensors –including ISFETs- for in-field monitoring is of special interest due to the portability and low power consumption provided compared with conventional sensors. Although their use is not extended, herein a review of the works published, including detection of surfactants and pesticides using ISFETs is detailed.

The analysis of surfactants in waters with ISFET-based sensors has been studied since the 90 ties, when the methodologies for measuring these compounds were improving due to the necessity of faster analysis and easier instrumentation for in-field analysis. Campanella *et al.* [72] used ISFETs with PVC membranes for detecting anionic and cationic surfactants. The main aspects of the developed sensors were their low detection limit compared with ISEs and the high selectivity against other anions.

Sanchez *et al.* [73] proposed the use of ISFETs for anionic and cationic surfactants determination using potentiometric titration and PVC based ISFETs. Calibrations for standard surfactants as sodium dodecylbenzenesulphonate (SDBS) and dodecylsulphate (SDS) resulted in near-nernstian slopes and quite low limits of detection (pLD around 6.4). Sensors were stable for four months under laboratory conditions but this lifetime decreased when real samples were used due to the membrane pealing. Authors suggest several applications particularly for the control of cleaning processes and routine analysis of industrial row.

Pesticides have been widely used in agriculture and, even though some regulations around the world prohibit the use, pesticides are still found in crops. The excess of pesticides is running away from soil to ground and surface waters thus presenting high toxicity for mammals in very low concentrations. Regulations are strictly enforced in all countries, with the maximum concentration permitted in drinking waters being 0.1 μg·L^−1^ by EU directives. Standard methods for measuring them are based on chromatographic techniques. Even though these are quite accurate and precise methods, they can not be applied in field and are costly and time consuming. Regarding the use of sensors for pesticide detection, the most common approach is the enzymatic inhibition methodology. Some pesticides, mainly organophosphorous and carbamates, inhibit the activity of enzymes from the cholinesterase family. A critical assessment outlining the advantages of using enzymatic ISFET sensors with acetylcholinesterase for pesticide determination was early reported by Janata *et al.* [74]. Later on, the group of Eniricerche [75,76] developed an ENFET with the enzyme acetilcolinesterase linked to the amino functional groups of a polysiloxane surface, obtaining a biosensor for paraoxon with good stability and reproducibility.

A differential ISFET-based system with immobilized cholinesterases (AcChE and BUChE) was reported by Hendji *et al.* [77] and applied to several kinds of organophosphorous pesticides. Special attention was given to the enzyme regeneration after exposure to the pesticide. This is a critical step since total regeneration is not usually achieved and dependence on membrane thickness and enzyme concentration is present. Other types of ENFETs using immobilization techniques in activated Nylon nets [78] and combining polymers like PVC and PVA have also been described [79,80]. Until now, none of these ISFET based sensors developed for pesticides monitoring have been applied to real samples due to the difficulties outlined.

The on-site measurement of parameters like pH, conductivity and oxidation-reduction potential (ORP) in waste waters is still not solved with conventional probes. These probes suffer from fouling, needing frequent maintenance and are quite expensive. Besides, the autonomy of these probes is low. In that context the use of microsensors can be advantageous due to the small size, lower power consumption and robustness. Recently, a versatile and portable system based on microsensors for measuring simultaneously pH, ORP, conductivity and temperature in wastewater samples was assembled and evaluated as reported in reference [81]. An ISFET for pH measurements and platinum microelectrodes for ORP and conductivity measurements were used within the system. The multi-parametric system included a commercial temperature sensor used to compensate, at the software level, the thermal drift of pH and conductivity. Wastewater samples coming from an industrial state collector were measured using this portable system. The results obtained for four pH-ISFETs in those samples showed a high sensor-to-sensor reproducibility with an average standard deviation of 2.4% for each sample. Comparison with data obtained from a commercial electrode provided a relative error between 3 and 5%, which is quite satisfactory for this kind of samples.

**Figure 5.**
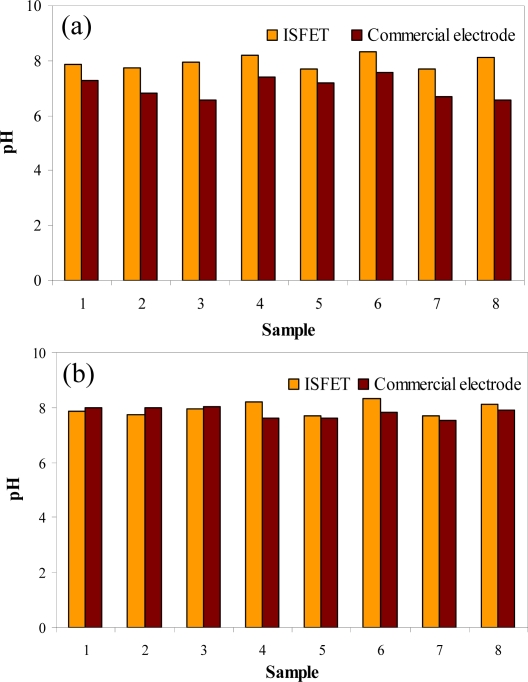
pH-ISFETs values of wastewater samples coming from a winemaking production line and comparison with those performed with a glass electrode. (a) Effluent. (b) Effluent after treatment.

This system was also used to measure pH of samples coming from a winemaking production line [81]. The effluents were interrogated after and before performing a conventional wastewater treatment during 8 days of sampling. pH values obtained before the treatment (Figure 5a) showed a slight variability due to the variable waste composition of the effluent during the 8 days the study lasted. However after the treatment (Figure 5b), values were more homogeneous. In the first case, relative errors obtained from the comparison between pH-ISFET data and glass electrode data were around 10%. However, errors were below 1.7% when measuring after effluent treatment. This fact might be explained by the high quantity of matter in suspension that is present in the untreated effluent that could affect the glass electrode response. The whole results clearly demonstrate the feasibility of using pH-ISFETs for wastewater monitoring.

### 3.2. Soils and Crops Measurements

Within the field of horticulture one of the major interests in sensor applications is for the control of drainage waters in crops. Chemical fertilizers are introduced in soils as essential nutrient sources for intensive agricultural production. The uncontrolled addition of these substances leads to runoff of nutrient excess into surface and ground waters causing an undesirable environmental impact (*i.e.*, the effects of nitrate lixiviation and the consequent nitrification of surface waters) as well as unnecessary increasing production costs. To sum up, this excess of nutrients may cause plant damage. Therefore, monitoring of nutrient levels in crops will provide useful information for reduction of environmental impact as well as for optimization of the overall production process. Additionally, if monitoring is carried out automatically and in real time, it would be possible to design intelligent irrigation and fertilization crop management systems.

Current standard methods for measuring ions in soil are based on sample treatment by means of extraction processes and analytical techniques like Atomic Absorption Spectroscopy and chromatographic techniques, which can not be used in-field and are time consuming. Therefore, the use of sensors opens the door to carry out the analysis *in situ* or on-line, thus results are obtained in real time. Currently, bibliography regarding the application of chemical sensors for soil measurements—that is not the same for physical sensors- is quite reduced. The potentialities of using the pH-ISFETs commercialized by Sentron for measuring soil samples using small volumes is outlined in reference [82]. The paper reports the results of pH and several ions measurement in samples extracted from the vicinity of roots of spruce trees. The study established a relationship between the effects of different chemical species present in soil with the tree growth properties.

**Figure 6.**
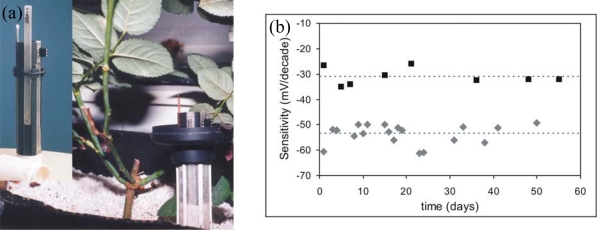
(a) Probe with a pH-ISFET and K^+^, Ca^2+^ and NO_3_ CHEMFETs and a reference electrode for measuring in soils. (b) Variation of sensors sensitivity inserted in a pot during two months (♦) potassium and (▪) calcium ISFETs.

A multiple sensor system with pH, K^+^, Ca^2+^ and NO_3_^−^ ISFET-based sensors was reported by Artigas *et al.* [83]. Those sensors and a reference electrode were implemented in a special probe applied directly in soil that permitted the easy handling of sensors and the protection of their sensitive area (see Figure 6a). Ionic membranes for ISFETs were based on photocurable polymers which provided long-term stability of the sensors. Those were stable over nine months in aqueous solutions and up to two months inserted in soil as shown in Figure 6b [26,84,85]. Analysis data of several extracted soil samples from rose pots with loam or pet soil were compared with those obtained with standard methods showing a good agreement between both methods.

A paper from Rossel *et al*. [86] confirms the usefulness of using ISFET sensors in precision agriculture to get maps of distribution. In that case, a quite detailed study of pH values in several sites of an agricultural field is performed with a pH-ISFET. A comparison between field pH data from ISFETs with data obtained in laboratory, considering effort and cost of each analysis, demonstrates the high efficiency of ISFETs and their practical application for measuring soil pH.

The use of artificial growing media is extended in almost all modern horticulture crops. Usually the fertilizer solution is supplied in liquid form and a closed system permits to reuse it. For a optimization of these systems it would be desirable to control the concentration of ions in the drainage water and compensate the uptake of ions by the plants by using a feedback controlled nutrient dispenser. This approach, using ISEs and CHEMFETs to monitor ions concentrations, is described in the reference [87]. Results are encouraging but some practical problems related with the lifetime of sensors are exposed. Following this idea of continuous monitoring of crops using ISFET-based sensors a flow system was reported by Van den Vlekkert *et al.* [88]. The system described included a flow-through cell with backside contacted ISFETs for pH and potassium measurement. A closed-loop system that monitored irrigation and drainage waters in hydrophonic substrates permitted to control plant growth and reuse drainage water. This system included also a temperature sensor for ISFET thermal drift correction and used a protocol for frequent calibration to minimize drift. Although results reported in this paper are not extensive, the robust packaging of the flow-through assembly by using BSC-ISFETs and the durability of potassium membranes was pointed out.

A flow injection method for real-time soil analysis was described by Birrel *et al.* [89]. This system incorporated an automated soil extraction apparatus and a multi-ISFET chip provided by Hitachi (Japan). Nitrate PVC membranes were developed for this system. Results were quite hopeful regarding fast response and reproducibility. However, non-linear responses were obtained in the concentration range of nitrates present in soil and slopes were lower than 40 mV/decade. A later study reported the results of nitrate estimation of soil cores using the same flow system and the automatic extraction apparatus [90].

### 3.3. Geochemical Barriers Monitoring

An innovative trend in environmental monitoring is the study of chemical and physical characteristics of geochemical barriers, placed in underground repositories to storage and isolate the nuclear waste from the biosphere [91]. Those repositories generally rely on a multi-barrier system that typically comprises the natural geological barrier provided by the repository host rock and its surroundings, and an engineered barrier system (EBS). The EBS represents the man-made, engineered materials placed within a repository, including the waste form, waste canisters, buffer materials, backfill and seals. Bentonite, a special kind of clay, is considered a suitable “buffer material” to be used as engineering barrier in a repository due to its low permeability, high swelling capacity and high plasticity [92,93].

The characterisation of the interstitial water in a geochemical barrier is crucial to evaluate the hydro-geochemical factors that control the transport and migration processes of radionuclides [94]. The composition of the pore water governs their solubility, affects on their sorption onto the mineral surfaces and the corrosion rate of the waste repositories [95]. Up to now, the different tests employed to monitor pore water in EBS are carried out *ex-situ*, in a laboratory. Consequently, samples are susceptible of alteration or degradation when taking them off from its natural medium. In this context, developing new systems for *in situ* monitoring of chemical parameters is of great interest [96,97].

**Figure 7.**
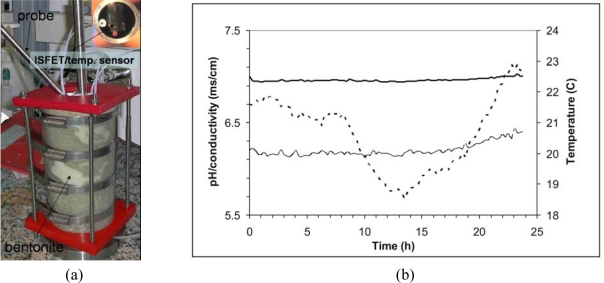
(a) Scale model with compacted bentonite and a stainless steel probe with sensors. (b) Response of pH-ISFET (**–**), conductivity (—) and temperature (---) sensors in the scale model during one day under continuous measurements.

pH, redox potential (ORP), and conductivity are some of the parameters used to give an insight into the chemical state of the system. A probe containing ISFET and Pt microelectrodes fabricated in the IMB-CNM for monitoring pore water of bentonite has been reported by Jimenez *et al.* [98,99]. The probe was fabricated with stainless steel to be inserted in a scale model with compacted bentonite (see Figure 7a). In order to compensate the temperature drift for the ISFET and conductivity sensors, a temperature sensor was also included. A multi-parametric system containing a commercial module with USB interface was used for the acquisition and control of data from a laptop PC [81]. As shown in Figure 7b the response of those sensors in the scale model during 25 h showed a high stability.

## 5. Conclusions

The introduction of ISFET-based sensors in different areas of analytical chemistry has prompted the scientific community to tackle new technological challenges for the implementation of sensors in more automatic and autonomous systems. Application of ISFET-based sensors to environmental monitoring, where miniaturization is not critical (sample is almost unlimited) appears to be of limited interest as a first option. However, there are very challenging analytical applications in this field as Table 2 reports. For example multiparametric detection probes, integrating various sensors on demand for *in situ* measurements are highly required for groundwater and marine water monitoring. It is in this context where ISFETs offer a highly added value due to their small size and robustness providing low power consumption. Besides, ISFETs are ideal for integration in (semi)automated flow systems (*i.e.*, FIA, SIA) or in miniaturized analytical systems (*i.e.*, μTAS, LoC) providing high throughput analysis, low reagent consumption and automatic sampling conditioning and calibration. Such benefits would be absolutely unexpected with conventional electrodes.

Nowadays, taking advantage of semiconductor based technology; other possibilities are being also explored. In this context, fabrication of sensor arrays that integrate different kind of silicon sensors such as ISFETs, metal electrodes and p-n diodes as temperature sensors have been performed. Such arrays, which constitute useful tools for multiparametric analysis by using miniaturized flow systems, are being implemented mostly in clinical diagnosis. However their feasibility in environmental monitoring is still a challenge. The use of wireless sensors is also a highly promising alternative to implement systems for in field applications (*i.e.*, extensive agriculture), which is nowadays being developed by many research groups and companies world wide.

## Figures and Tables

**Table 1.** Electrical and chemical characteristics measured for a complete wafer (n = 413)*.

	**Electrical Parameters**			**Chemical Parameters**		

	V_out_ (mV at pH 7)	V_th_ (mV)	Gm (μAV^−2^)	Sensitivity (mVpH^−1^)	E_0_ (mV)	R^2^
Mean Value	316.9	−120.1	74.0	57	−47	0.9995
Stand. Deviation	155.2	154.2	2.5	2	163	
Max. Value	791.6	434.0	82.0	59	406	0.9999
Min. Value	−131.2	−552.0	62.9	52	−523	0.9984

* nº of total devices 450; Rejected: 37; Yielding 82%

**Table 2.** Summary of ISFETs applied in the environmental field.

**ISFET type or system^a^**	**Measured parameter**	**Sample or application**	**Reference**
ISFET (PVC-PVA-PVAc membrane)	NO_3_^−^	river water and soil extracts	[58]
ISFET /CHEMFET	pH, NO_3_^−^	acid-rain	[59]
ISFET	pH	rain droplets	[60]
ISFETs/CHEMFETs (siloprene membrane)	pH and NH_4_^+^, NO_3_^−^	Water/general purpose	[62]
ISFET (PVC membrane)/FI system	pH, NH_4_^+^	water / general purpose	[65]
BSC-ISFET (PVC membrane)/CF system	NH_4_^+^	water / general purpose	[66]
BSC-ISFET (PVC membrane)/FI system	pH, NH_4_^+^, Ca^2+^, NO_3_^−^	water / general purpose	[67]
pH-ISFET	acidification rates	water (micro-organisms)	[68]
ISFET/flow system	smart living space	drinking and swimming pool water	[69]
Multisensor system	pH, Cl^−^	general purpose	[70]
pH-ISFET/smart system	pH	general purpose	[71]
ISFET(PVC membrane)	surfactants	wastewater	[72]
CHEMFET(PVC membrane)/titration	surfactants	wastewater	73]
AcChE ENFET	pesticides	Wastewater/general purpose	[74]
AcChE ENFET	paraoxon	Wastewater/general purpose	[75,76]
AcChE BuChE ENFET/differential system	pesticides	Wastewater/general purpose	[77]
AcChE ENFET (Nylon membrane)	pesticides	wastewater	[78]
AcChE ENFET (PVC-PVA membrane)	pesticides	Wastewater/general purpose	[79,80]
pH-ISFET/mutiparametric system	pH (ORP, conductivity)	wastewater	[81]
pH-ISFET	pH	soil extracts	[82]
ISFET-CHEMFET probe (photocurable membranes)	pH, K^+^, Ca^2+^, NO_3_^−^	in-soil	[83]
pH-ISFET	pH	in-soil	[86]
CHEMFET	K^+^, Ca^2+^, NO_3_	Horticulture drainage water	[87]
BSC- ISFET/flow system	pH, K^+^	hydrophonic substrates	[88]
CHEMFET (PVC membrane)/flow system	NO_3_^−^	soil extracted samples	[89,90]
multiparametric probe	pH, ORP, conductivity	geochemical barriers	[98,99]

a BSC, Back side contact; FI, flow injection; CF, continuous flow; AcChE, acetylcholinesterase; BuChE, butil cholinesterase.
